# Lymph Node Dissection Guideline Adherence and Survival in Patients With T1N0M0 Lung Adenocarcinoma

**DOI:** 10.1001/jamaoncol.2025.5924

**Published:** 2026-01-15

**Authors:** Renda Li, Pan Wang, Hao Zhang, Qingpeng Zeng, Chenran Wang, Ni Li, Wenjing Yang, Fengwei Tan, Jie He

**Affiliations:** 1 Department of Thoracic Surgery, National Cancer Center/National Clinical Research Center for Cancer/Cancer Hospital, Chinese Academy of Medical Sciences and Peking Union Medical College, Beijing, China; 2 Office of Cancer Screening, National Cancer Center/National Clinical Research Center for Cancer/Cancer Hospital, Chinese Academy of Medical Sciences and Peking Union Medical College, Beijing, China; 3 Office for Cancer Diagnosis and Treatment Quality Control, National Cancer Center/National Clinical Research Center for Cancer/Cancer Hospital, Chinese Academy of Medical Sciences, Peking Union Medical College, Beijing, China

## Abstract

**Importance:**

Lymph node dissection for early-stage lung adenocarcinoma is controversial. Histologic pattern subtyping reveals heterogeneity of lung adenocarcinoma, yet its association with lymph node involvement and dissection is understudied.

**Objective:**

To assess the association between guideline-adherent lymph node dissection, histologic pattern subtyping, and overall survival in patients with clinical T1N0M0 lung adenocarcinoma.

**Design, Setting, and Participants:**

This multicenter cohort study used data from the National Cancer Center LungReal database, a multicenter, electronic health records-based database for patients undergoing surgery for lung cancer, from January 2014 to December 2021, with the last follow-up in December 2022. Patients were categorized based on histologic pattern of adenocarcinoma into 2 groups: lepidic without high-grade pattern, and high-grade or no lepidic pattern. The data analysis was performed from April to November 2025.

**Exposure:**

Lymph node dissection adherence or nonadherence with the 3 + 1 standard (3 N2 plus 1 N1 station) and the 6-station standard (the subcarinal plus 2 other N2 stations and 3 N1 stations).

**Main Outcome and Measure:**

Overall survival.

**Results:**

Of 35 265 patients screened, 27 191 participants (mean [SD] age, 58.3 [11.7] years; 16 280 female [59.9%] and 10 911 male [40.1%] individuals) from 19 centers were included; among them, 15 593 (57.3%) received lymph node dissection adherent with the 3 + 1 standard and 4023 (14.8%) with the 6-station standard. Among the group of 13 369 patients (49.2%) with adenocarcinoma of lepidic without high-grade pattern, no association was observed between survival and adherence with the 3 + 1 standard (hazard ratio [HR], 0.81; 95% CI, 0.57-1.15) or the 6-station standard (HR, 0.54; 95% CI, 0.26-1.13). Whereas, among the group of 13 822 patients (50.8%) with adenocarcinoma of high-grade or no lepidic pattern, adherence with the 3 + 1 standard (HR, 0.81; 95% CI, 0.69-0.95; absolute risk difference at 3-year, 1.2%; 95% CI, 0.2%-2.2%; E-value, 1.78) or the 6-station standard (HR, 0.61; 95% CI, 0.45-0.83; absolute risk difference at 3 years, 1.0%; 95% CI, 0.1%-1.9%; E-value, 2.67) was associated with a significant but small absolute survival benefit.

**Conclusion and Relevance:**

In this cohort study, guideline-adherent nodal dissection was associated with small absolute survival benefit for patients with adenocarcinoma of high-grade or no lepidic pattern, but not for those with lepidic without high-grade pattern. These observational findings warrant prospective validation and should not be interpreted as causal.

## Key Points

**Question:**

Is guideline-adherent lymph node dissection associated with survival benefit among patients with cT1N0M0 lung adenocarcinoma?

**Findings:**

In this cohort study of 27 191 patients with cT1N0M0 lung adenocarcinoma from a multicenter clinical database, lymph node dissection adherent with the 3 + 1 or the 6-station standard was associated with survival benefit among patients with adenocarcinoma of high-grade or no lepidic pattern, but not with lepidic without high-grade pattern.

**Meaning:**

These findings suggest that the survival impact of guideline-adherent lymph node dissection differed among patients with adenocarcinomas of different histologic pattern types.

## Teaser

This multicenter cohort study assesses the association between guideline-adherent lymph node dissection, histologic pattern subtyping, and overall survival in patients with clinical T1N0M0 lung adenocarcinoma.

## Introduction

The extent of lymph node dissection for early-stage lung cancer is evolving and is a subject of long debate. Lymph node dissection standards recommended by current clinical guidelines were inconsistent. A 3 + 1 standard of lymph node dissection, which requires assessment of a minimum of 1 hilar or intrapulmonary N1 station and 3 mediastinal N2 stations, is recommended by the US National Comprehensive Cancer Network guideline^1^ and the American College of Surgeons Commission on Cancer Operative Standard 5.8.^2^ A more stringent 6-stations lymph node dissection standard, which requires assessment of at least 6 nodal stations including the subcarinal station plus 2 other N2 stations and 3 N1 stations, is recommended by the International Association for the Study of Lung Cancer^3^ and the Chinese Society of Clinical Oncology non−small cell lung cancer guideline.^4^ Conflicting results have been reported on whether improved survival is associated the 3 + 1 standard^5,6,7^ or the 6-station standard.^8,9^ Notably, conflicting data have been predominantly observed in patients with clinical stage I lung cancer,^5,9^ indicating a gap of knowledge for optimized lymph node dissection for patients with early-stage lung cancer.

The establishment of histologic subtyping has led to the recognition of heterogeneity of lung adenocarcinomas. Lepidic pattern has been reported to be associated with low risk of lymph node involvement, disease recurrence, and death.^10,11,12^ Histologic features, including micropapillary, solid, complex glandular, and cribriform patterns, were associated with more advanced N classification^12,13^ and elevated risk of disease recurrence and death.^11,12^ Need for risk-based adjustments of lymph node dissection according to histologic subtyping were proposed.^9,10,14^ However, whether histologic pattern should be taken into consideration in the extent of lymph node dissection remain unclear, with few supporting data. In addition, the success of performing an effective lymph node dissection lies in the firm knowledge of lymph node involvement pattern. Updates of lymph node involvement pattern integrating histologic subtyping of adenocarcinomas are required.

Aiming to fulfill these unmet needs, we performed comprehensive analyses on lymph node involvement and survival associated with lymph node dissection guidelines using a multicenter clinical database, the National Cancer Center (NCC) LungReal database. Histologic pattern subtyping and size were integrated into the analyses of lymph node involvement and the association of survival with guideline adherence. We hope to provide fundamental data on lymph node involvement, and to provide evidence for recent concern on lymph node dissection for early-stage lung adenocarcinomas.

## Methods

This study was part of the NCC LungReal study, a multicenter, electronic health records−based clinical study of patients who underwent surgery for lung cancer. The NCC LungReal study was approved by NCC ethics committee (No. 19/217-2001) and was registered in ClinicalTrials.gov (NCT06255197). Written informed consent was waived due to the noninterventional nature of this study and the use of de-identified data. The data used were from study phase 1, release a09. We followed the Strengthening the Reporting of Observational Studies in Epidemiology (STROBE) reporting guideline.

### Study Population

Patients who were diagnosed with clinical T1N0M0 lung cancer—per the *TNM Classification of Malignant Tumors*, eighth edition—and who underwent surgical resection with pathologically confirmed invasive adenocarcinoma from January 2014 to December 2021 were screened for study entry. We excluded patients diagnosed with minimally invasive adenocarcinomas or receiving neoadjuvant therapy; with intrathoracic metastasis discovered during surgery, R1 or R2 residual disease, or synchronous multiple primary tumors; who did not undergo lymph node dissection; had no data on adenocarcinoma histologic patterns; or whose baseline characteristics or survival data were incomplete.

### Description of Lymph Node Assessment and Involvement

The study population was stratified into 2 groups according to each standard, resulting in 4 subgroups overall: adherent with 3 + 1 standard, nonadherent with 3 + 1 standard, adherent with 6-station standard, or nonadherent with 6-station standard. However, comparative analyses were conducted only between the adherent and nonadherent groups for each respective standard. Nodal stations were named according to International Association for the Study of Lung Cancer nodal definitions. The involvement rate for station *K* was calculated as the number of cases with station *K* nodes involved divided by the number of cases with station *K* nodes dissected and examined. To avoid distortion by outliers when a sample size is small, we did not present the involvement rate when a given station was dissected and examined in fewer than 30 patients.

### Category of Adenocarcinoma by Size and Histologic Patterns

We categorized adenocarcinomas by increments of 1 cm by the largest diameter. Each interval included the upper limit but not the lower limit (eg, 2- to 3-cm diameter indicates >2 cm and ≤3 cm).

The presence or absence of major histologic patterns of lung adenocarcinoma (lepidic, acinar, papillary, micropapillary, solid, complex glandular, and cribriform) were recorded in the NCC LungReal study phase 1, release a09 data. Adenocarcinomas were categorized into 1 of 2 histologic pattern groups: (1) lepidic with no high-grade (LepNH) pattern, defined as tumors with a lepidic pattern and none of the following 4 high-grade patterns: solid, micropapillary, complex glandular, or cribriform; or (2) high-grade or no lepidic (HGNL) pattern, defined as tumors with any of the 4 high-grade patterns or without lepidic pattern.

### Statistical Analysis

Comparisons were performed using χ^2^ test or Wilcoxon rank sum test where appropriate. Relative risk (RR) and its 95% CIs of nodal involvement associated with histologic pattern were estimated using a marginal standardization of a logistic model. Missing data were multiply imputed using chained equations.

Overall survival (OS) was the primary outcome and was defined as the interval between surgery and death or last follow-up. Survival was estimated using Kaplan-Meier method. The primary comparison was the comparison between the adherent and the nonadherent groups in the overall HGNL group. Hazard ratio (HR) and 95% CIs associated with lymph node dissection guideline adherence were estimated using Cox proportional hazard regression models within each imputed dataset. These models included relevant variables (ie, age at diagnosis, sex, smoking history, year of surgery, surgery method, lung resection extent, and lobe of origin), and a hospital-specific shared-frailty random effect to account for hospital-level clustering.^15^ Results from each imputed dataset were pooled via Rubin rules. Absolute risk difference, number needed to treat, restricted mean survival time (RMST) and RMST difference were calculated according to standard methods.^16^ We performed exploratory analyses of comparisons between the adherent and nonadherent groups in subgroups stratified by 1-cm increments in tumor size, and between adherence to either the more stringent 6-station standard or only the 3 + 1 standard in subgroups in which guideline adherence was associated with survival benefit. In analysis with fewer than 10 events per variable, results should be interpreted as exploratory because small number of events affected the accuracy of HR and CI estimation.^17^

Inverse probability of treatment weighting was performed to balance baseline covariates, with truncated-weight and doubly robust augmented inverse probability weighting sensitivity analyses. Multiple sensitivity analyses were performed. Further details are provided in the eMethods in Supplement 1.

All tests were 2-sided with a *P* < .05 defined as significant. All statistical analyses were performed from April to November 2025 with R, version 4.4.2 (R Foundation for Statistical Computing).

## Results

A total of 35 265 patients who were clinically diagnosed with T1N0M0 lung cancer, underwent surgical resection, and had pathologically confirmed invasive adenocarcinoma between January 2014 and December 2021 were identified from the NCC LungReal database. Of these, 27 191 patients (mean [SD] age, 58.3 [11.7] years; 16 280 female [59.9%] and 10 911 male [40.1%] individuals) from 19 centers were included in the analyses (eFigure 1 in Supplement 1 provides the flow diagram). In all, 15 593 patients (57.3%) received lymph node dissection adherent with the 3 + 1 standard, and 4023 patients (14.8%) received lymph node dissection adherent with the 6-station standard. When adenocarcinomas were categorized by histologic patterns, the LepNH group comprised 13 369 patients (49.2%) and the HGNL group, 13 822 patients (50.8%). Valid follow-up data were available for all included patients from the last round of follow-up conducted between September and December 2022. By the end of the follow-up period, 798 patients (2.9%) had died. The median (IQR) follow-up time for living patients was 24.9 (12.3-37.1) months.

The baseline characteristics of patients based on adherence with lymph node dissection guidelines are summarized in the Table. Notably, pathologic up-staging to pathologic N1-2 category was more frequently observed in patients who received lymph node dissection adherent with the 3 + 1 standard (10.2% vs 5.7%) or the 6-station standard (10.3% vs 7.9%).

**Table.** Baseline Characteristics of Patients Receiving Lymph Node Dissection Adherent or Not Adherent With Guideline Standards

Characteristic	Guideline, No. (%)					
	3 + 1 Standard			6-Station standard		
	Adherent	Nonadherent	*P* value	Adherent	Nonadherent	*P* value
Participants, No.	15 593	11 598	NA	4023	23 168	NA
Age, median (IQR), y	59 (52-66)	60 (52-66)	<.001	58 (51-65)	60 (52-66)	<.001
Sex						
Female	9367 (60.1)	6913 (59.6)	.40	2363 (58.7)	13 917 (60.1)	.11
Male	6226 (39.9)	4685 (40.4)		1660 (41.3)	9251 (39.9)	
Smoking history						
Never	12 093 (77.6)	8663 (74.7)	<.001	2988 (74.3)	17 768 (76.7)	<.001
Current/ever	3500 (22.4)	2935 (25.3)		1035 (25.7)	5400 (23.3)	
**Surgical history**						
Primary tumor size, median (IQR), cm	1.7 (1.3-2.3)	1.5 (1.1-2.0)	<.001	1.7 (1.3-2.2)	1.6 (1.2-2.2)	<.001
Preoperative PET-CT^a^	1392 (8.9)	1030 (8.8)	.89	366 (9.1)	2056 (8.8)	.65
Surgery method						
VATS	14 686 (95.3)	10 963 (95.8)	<.001	3840 (96.3)	21 809 (95.4)	.02
Open surgery	525 (3.4)	438 (3.8)		117 (2.9)	846 (3.7)	
RATS	195 (1.3)	48 (0.4)		29 (0.7)	214 (0.9)	
Missing data	187	149		37	299	
Lung resection extent						
Lobectomy	13 176 (86.6)	7989 (70.4)	<.001	3404 (85.9)	17 761 (78.6)	<.001
Segmentectomy	1189 (7.8)	1808 (15.9)		411 (10.4)	2586 (11.4)	
Wedge resection	807 (5.3)	1456 (12.8)		141 (3.6)	2122 (9.4)	
Extensive resection	47 (0.3)	90 (0.8)		5 (0.1)	132 (0.6)	
Missing data	374	255		62	567	
Station dissected, median No. (IQR)						
N1	2 (1-3)	1 (1-2)	<.001	3 (3-3)	2 (1-2)	<.001
N2	3 (3-4)	2 (1-2)	<.001	3 (3-4)	3 (2-3)	<.001
Total	6 (5-6)	3 (2-4)	<.001	6 (6-7)	4 (3-5)	<.001
Node dissected, median (IQR)						
N1	3 (2-5)	2 (1-4)	<.001	5 (4-7)	2 (1-4)	<.001
N2	7 (4-10)	2 (1-5)	<.001	6 (4-10)	5 (2-8)	<.001
Total	11 (8-15)	5 (3-8)	<.001	12 (9-17)	7 (5-12)	<.001
**Disease characteristics**						
Histologic pattern group						
LepNH	7051 (45.2)	6318 (54.5)	<.001	2031 (50.5)	11 338 (48.9)	.07
HGNL	8542 (54.8)	5280 (45.5)		1992 (49.5)	11 830 (51.1)	
Pathologic T category^b^						
T1	13 227 (84.8)	10 197 (87.9)	<.001	3494 (86.9)	19 930 (86.0)	.30
T2	2341 (15.0)	1388 (12.0)		525 (13.0)	3204 (13.8)	
T3-4	25 (0.2)	13 (0.1)		4 (0.1)	34 (0.1)	
Pathologic N category^b^						
N0	14 004 (89.8)	10 931 (94.2)	<.001	3609 (89.7)	21 326 (92.0)	<.001
N1	518 (3.3)	280 (2.4)		161 (4.0)	637 (2.7)	
N2	1071 (6.9)	387 (3.3)		253 (6.3)	1205 (5.2)	
Pathologic TNM stage^b^						
IA	12 233 (78.5)	9811 (84.6)	<.001	3199 (79.5)	18 845 (81.3)	<.001
IB	1771 (11.4)	1120 (9.7)		410 (10.2)	2481 (10.7)	
IIB	517 (3.3)	280 (2.4)		160 (4.0)	637 (2.7)	
III	1072 (6.9)	387 (3.3)		254 (6.3)	1205 (5.2)	

Abbreviations: HGNL, adenocarcinomas of high-grade pattern or no lepidic pattern; LepNH, adenocarcinomas of lepidic and no high-grade pattern; PET-CT, positron emission tomography-computed tomography; NA, not applicable; RATS, robotic-assisted thoracoscopy surgery; VATS, video-assisted thoracoscopic surgery.

^a^ Defined as within 90 days before the date of surgery.

^b^ Defined according per the *TNM Classification of Malignant Tumors*, eighth edition.

Percentages may not add up to 100% due to rounding.

### Association of Histologic Pattern and Tumor Size With Lymph Node Involvement

**Figure 1.**
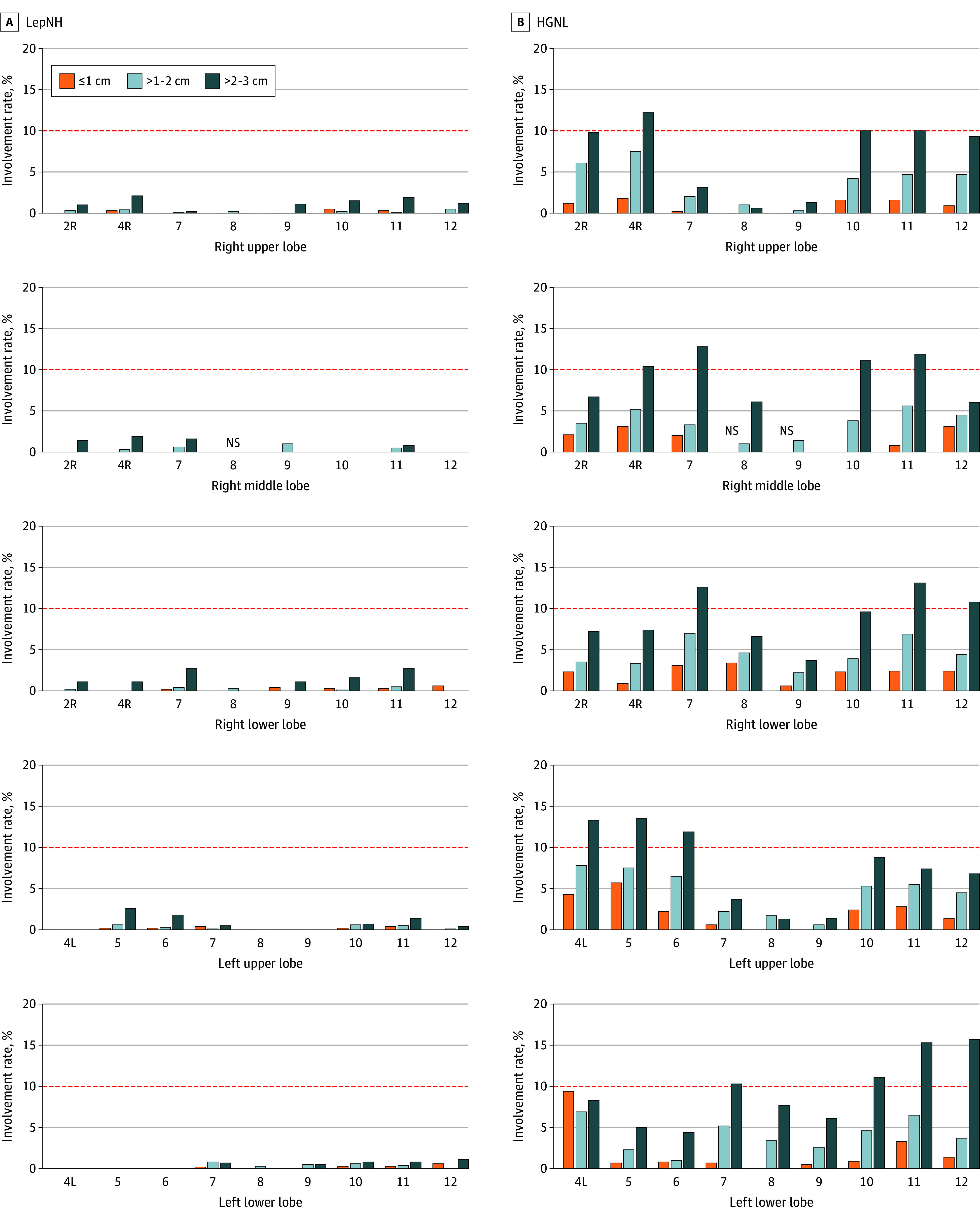
Involvement Rate of Each Nodal Station by Lung Adenocarcinomas A, Lipidic with no high-grade (LepNH) adenocarcinomas. B, High-grade or no lepidic (HGNL) adenocarcinomas, stratified by lobe of origin and 1-cm increments of size. The red dashed horizontal line denotes the level of 10%. Stations 3, 13, and 14 are not present due to low count. Bars indicating a station dissected in fewer than 30 patients are not shown to avoid outliers when sample size was small. NS indicates not shown.

We generated a lobe of origin, size, and histologic pattern-specific summary of lymph node involvement rates for each nodal station (Figure 1 and eTables 1 and 2 in Supplement 2. Sharp differences in involvement rates were observed between the LepNH group and the HGNL group. The nodal involvement rates for adenocarcinomas among the LepNH group were low. For the adenocarcinomas 2-cm or smaller in the LepNH group, the maximal involvement rate for mediastinal stations and hilar or intrapulmonary stations was 1.0% and 0.6%, respectively. Involvement rates reached up to 2.7% only in patients with 2- to 3-cm adenocarcinomas in the LepNH group (eTable 1 in Supplement 2). On the contrary, nodal involvement rates for adenocarcinomas in the HGNL group were considerably high: for 2- to 3-cm adenocarcinomas, maximal involvement rates by tumors from each lobe of origin ranged from 10.3% to 13.5% for mediastinal stations, and 8.8% to 15.7% for hilar or intrapulmonary stations (eTable 2 in Supplement 2).

Except for station 4L, 8, and 9, which appeared to be outliers given the small number of patients involved, the HGNL group adenocarcinomas were associated with RRs of involvement ranging from 8.5 to 19.8 compared to the LepNH group in each nodal station (eTable 3 in Supplement 2). The increase of 1 cm in maximal diameter of primary tumor was associated with RRs for nodal involvement ranging from 1.36 to 2.99 in each nodal station (eTable 4 in Supplement 2).

### Association of Histologic Pattern Group With Survival

The 3-year OS for patients with adenocarcinomas in the LepNH group vs HGNL group was 98.7% and 94.2%, respectively (eFigure 2 in Supplement 1). After adjustment, the HGNL group adenocarcinomas were associated with a significantly increased risk of mortality (HR, 2.10; 95% CI, 1.71-2.56; *P* < .001).

### Association of Guideline Adherence With OS by Histologic Pattern Group

#### LepNH Group

**Figure 2.**
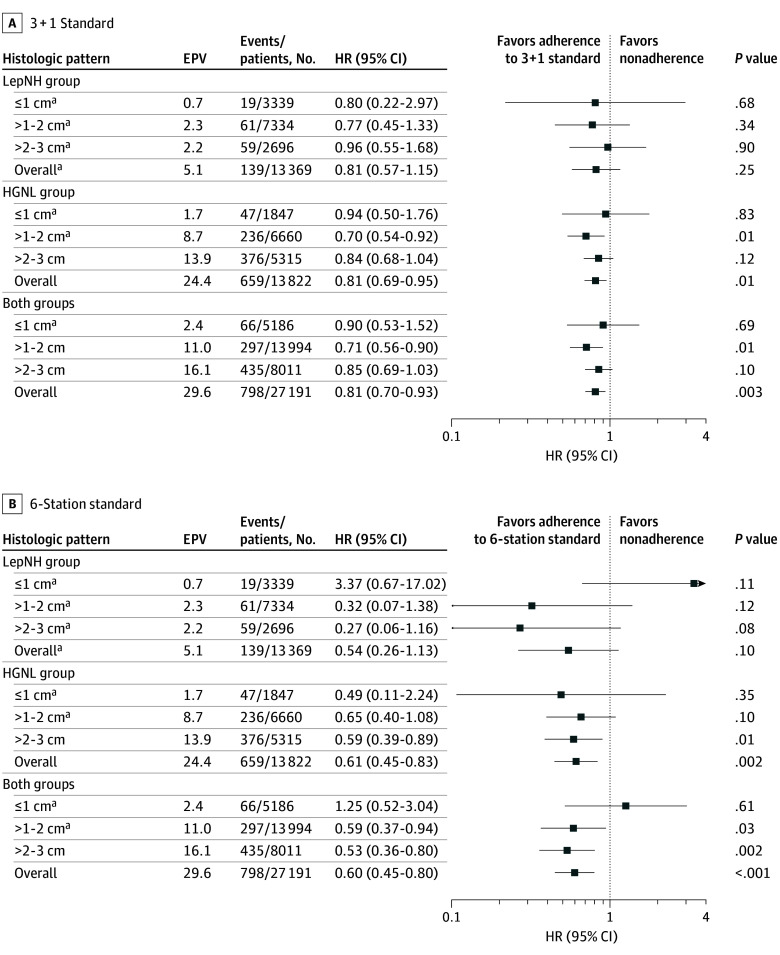
Association of Survival with Lymph Node Dissection Adherence or Nonadherence With Guideline Standard Overall refers to all patients in the histologic pattern category, irrespective of size. Right-sided arrowheads indicate upper limits of 95% CI >4. EPV indicates events per variable; LepNH, lepidic with no high-grade; HGNL, high-grade or no lepidic group; HR, hazard ratio. ^a^EPV <10. Results from subgroups with EPV <10 should be interpreted as exploratory.

Among patients with adenocarcinoma in the LepNH group, lymph node dissection adherent with the 3 + 1 standard (HR, 0.81; 95% CI, 0.57-1.15; *P* = .25) or the 6-station standard (HR, 0.54; 95% CI, 0.26-1.13; *P* = .10) was not associated with survival benefit (Kaplan-Meier survival curves are presented in eFigure 3 in Supplement 1). Exploratory analyses showed that the lack of association between survival benefit and guideline adherence persisted in the 1-cm or smaller, 1- to 2-cm, and 2- to 3-cm adenocarcinoma subgroups of patients in the LepNH group (Figure 2).

Good long-term survival was achieved irrespective of guideline adherence among patients with adenocarcinoma in the LepNH group. Three-year OS for the adherent or the nonadherent group ranged from 97.5% to 99.7% in the 1-cm or smaller adenocarcinoma subgroup, from 98.5% to 99.8% in the 1- to 2-cm subgroup, and from 96.6% to 99.7% in 2- to 3-cm subgroup (eTable 5 in Supplement 2). It should be noted that counts of outcome events were few in patients with adenocarcinoma in the LepNH group (139 of 13 369 patients), rendering results exploratory.

#### HGNL Group

Among patients with adenocarcinoma in the HGNL group, significant survival benefit was associated with lymph node dissection adherent with the 3 + 1 standard (HR, 0.81; 95% CI, 0.69-0.95; *P* = .009; absolute risk difference at 3 years, 1.2%; 95% CI, 0.2%-2.2%; E-value, 1.78; detailed results in eTable 6 in Supplement 2) or dissection adherent with the 6-station standard (HR, 0.61; 95% CI, 0.45-0.83; *P* = .002; absolute risk difference at 3 years, 1.0%; 95% CI, 0.1%-1.9%; E-value, 2.67). The RMST for the 3 + 1 standard adherent vs nonadherent groups was 35.4 (95% CI, 35.3-35.5) months and 35.0 (95% CI, 34.9-35.2) months at 3 years, respectively, with an RMST difference of 0.3 (95% CI, 0.2-0.5) months. The RMST for the 6-station standard adherent vs nonadherent groups was 35.6 (95% CI, 35.4-35.8) months and 35.1 (95% CI, 35.0-35.2) months at 3 years, respectively, with an RMST difference of 0.5 (95% CI, 0.3-0.7) months. Inverse probability of treatment weighted multivariable Cox regression models and sensitivity analyses revealed similar results (detailed in the eMethods and eResults in Supplement 1 and eTables 7 and 8 in Supplement 2).

Exploratory analysis in subgroups stratified by 1-cm increments in size of adenocarcinoma showed that the association between lymph node dissection adherent to the 3 + 1 standard and survival benefit persisted in the 1- to 2-cm subgroup (HR, 0.70; 95% CI, 0.54-0.92; *P* = .01; absolute risk difference at 3-year, 2.2%; 95% CI, 0.6%-3.8%; E-value, 2.20; Figure 2). The association between adherence with the 6-station standard and survival benefit persisted in the 2- to 3-cm subgroup (HR, 0.59; 95% CI, 0.39-0.89; *P* = .01; absolute risk difference at 3-year, 1.1%; 95% CI, 0.2%-2.0%; E-value, 2.80). Outcome events were sparse in the HGNL group for the 1-cm or smaller subgroup (47 of 1847 patients) and the 1- to 2-cm subgroup (236 of 6660), rendering results from these subgroups exploratory.

#### Association of 6-Station Standard Adherence With Survival

**Figure 3.**
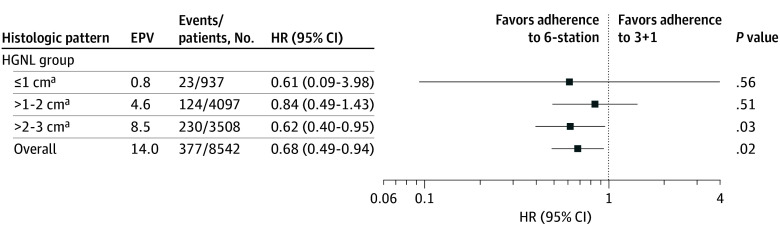
Exploratory Analysis of Association of Survival With Lymph Node Dissection Adherent With Only the 3 + 1 Standard or the 6-Station Standard Overall refers to all patients in the histologic pattern category irrespective of size. EPV indicates events per variable; HGNL, high-grade or no lepidic group; HR, hazard ratio. ^a^EPV <10. Results from subgroups with EPV <10 should be interpreted as exploratory.

Exploratory comparisons between lymph node dissections adherent with different standards was performed among patients with adenocarcinoma in the HGNL group. Among patients with 2- to 3-cm adenocarcinoma in the HGNL group, lymph node dissection adherent with the more stringent 6-station standards was associated with better survival (HR, 0.62; 95% CI, 0.40-0.95; *P* = .03; absolute risk difference at 3 years, 0.9%; 95% CI, 0.1%-1.7%; E-value, 2.63; Figure 3; eTable 9 in Supplement 2) than that adherent solely with the 3 + 1 standard.

## Discussion

Leveraging the magnitude and granularity of a multicenter clinical database for patients with surgically resected lung cancer, to our knowledge, this study presents the largest analysis of survival associated with lymph node dissection for patients with cT1N0M0 lung adenocarcinoma. We revealed heterogeneity of lung adenocarcinoma according to histologic pattern, featured by distinct levels of risks for lymph node involvement and mortality. We further analyzed survival associated with lymph node dissection guideline adherence in subgroups based on histologic pattern. These data offered insight into the long-debated area of lymph node dissection for early-stage lung adenocarcinoma and warranted further optimization of lymph node dissection using prospective data.

A rigorous lymph node dissection may bring survival benefit by removing potential metastases in lymph nodes, providing accurate pathologic N staging and directing patients with lymph node involvement to beneficial adjuvant therapies. However, lymph node dissection is also associated with increased risk of complications, including bleeding, chylothorax, nerve injury, and bronchopleural fistula.^18,19^ Recent studies suggest that the draining of lymph nodes plays an important role in antitumor immunity.^20,21^ Hence, more survival benefit is anticipated with extensive lymph node dissection in patients at higher risk for lymph node involvement. On the contrary, performing extensive lymph node dissection for patients who are at low or no risk of lymph node involvement could hardly introduce additional survival benefit.

We found that patients with clinical T1N0M0 adenocarcinomas in the LepNH group were at low risk for lymph node involvement and had excellent long-term survival. More limited lymph node dissection than guideline standards was not associated with significantly compromised OS in this study. In a prospective study by Zhang et al,^22^ lepidic-predominant adenocarcinoma on intraoperative frozen section accurately predicted negativity of mediastinal nodes. Yu et al^23^ reported a zero percentage of pathologic N1-2 disease in 133 patients with lepidic-predominant adenocarcinoma of 3 cm or smaller. Consequently, Cheng et al^10^ compared patients with lepidic-predominant adenocarcinoma who had 3 or more mediastinal stations assessed (139 patients) or more limited mediastinal lymph node dissection (82 patients) and found no significant difference in 5-year OS (99.3% and 98.7%, respectively). A more limited lymph node dissection for adenocarcinomas with lepidic-predominant pattern was proposed by several studies.^10,22,24^ However, the excellent long-term survival and sparse mortality events rendered statistical testing in most studies, including this one, not confirmatory.

In this study, HGNL group adenocarcinomas were associated with approximately 10 times higher risks for lymph node involvement and approximately 2 times higher risks for mortality than LepNH group adenocarcinomas. Among patients with adenocarcinomas in the HGNL group, adjusted estimates suggest an association between guideline-adherent lymph node dissection and survival benefit. However, absolute risk differences at 3 years were small (approximately 1 percentage point; number needed to treat, approximately 80-100), with confidence intervals in exploratory subgroups that often approach the null. The presence of high-grade pattern in lung adenocarcinoma was long recognized as a factor for worse survival and more advanced lymph node involvement.^11,12,23^ Sun et al^25^ also discovered that systemic mediastinal lymphadenectomy was an independent factor for favorable OS than limited mediastinal lymphadenectomy in patients with total percentage of micropapillary plus solid component greater than 5%, with an HR of 0.51. Our findings support selective application of guideline-adherent lymph node dissection in the HGNL group. However, given the observational design, potential residual confounding (including unmeasured pre-operative staging and treatment factors), sparse events in some strata, and modest E-values, these estimates should be viewed as associations of modest magnitude. Results should not be viewed as a universal mandate and require prospective confirmation.

The high-grade pattern of lung adenocarcinoma could be discerned by intraoperative frozen section. Multiple studies reported only moderate sensitivity ranging from 37% to 69% of intraoperative frozen section to discover high-grade pattern,^26,27,28^ indicating that a considerable proportion of HGNL group adenocarcinomas would be misclassified. Perioperative parameters, especially radiomics features, were reported to have high performance to predict high-grade patterns.^29,30^ Future studies are warranted to develop accurate methods to discover high-grade patterns intraoperatively or even pre-operatively.

### Limitations

First, the retrospective nature of NCC LungReal study was associated with unmeasured and unrecognized confounders that may contribute to both lymph node dissection selection and OS. Second, further maturation of survival data was anticipated. Number of outcome events was still limited for some subgroups. We expect to update the results during future follow-up of NCC LungReal cohort. Third, outcomes of disease recurrence, which are more sensitive for early-stage lung cancer, were not available in this study. Fourth, we lacked endobronchial ultrasonography or mediastinoscopy and adjuvant therapy data; both could influence adherence and outcomes. Although balance was achieved on observed covariates, unmeasured confounding cannot be excluded. We provide weight truncation, effective sample size, and doubly robust sensitivity analyses to assess robustness. Finally, results of this study should be interpreted as hypothesis generating. Unlike randomized clinical trials in which patients are required to be eligible for all lymph node dissection extents, limited lymph node dissection (more limited than either of the 2 standards considered) may have been selected by surgeons as a compromise in patients who were medically intolerant of extensive dissection. Factors associated with this compromise could not be collected in the clinical setting and could not be mitigated despite robust statistical adjustment.

## Conclusions

In this cohort study conducted in the clinical practice setting, lymph node dissection that adhered with the 3 + 1 standard or the 6-station standard was associated with survival benefit among patients with cT1N0M0 lung adenocarcinoma of high-grade or no lepidic pattern. However, neither standard of lymph node dissection was associated with survival benefit among those with adenocarcinoma of the lepidic without high-grade pattern. These observational findings warrant prospective validation and should not be interpreted as causal.

## Notes

Supplement 1. eMethods eResults eFigure 1. Diagram of the study population selection steps eFigure 2. Survival curves by histologic pattern group of lung adenocarcinoma eFigure 3. Survival curves by compliance to lymph node dissection guideline eFigure 4. Directed acyclic graph eFigure 5. Absolute standardized mean difference before and after weighting eFigure 6. Histograms of stabilized weights eReferences

Supplement 2. eTable 1. Involvement rate for eah nodal station by LepNH group adenocarcinoma with different lobe-of-origin and size eTable 2. Involvement rate for eah nodal station by HGNL group adenocarcinoma with different lobe-of-origin and size eTable 3. Relative risks of involvement in each nodal station associated with histologic pattern grouping of lung adenocarcinoma eTable 4. Relative risks of involvement in each nodal station associated with 1cm increase in size of lung adenocarcinoma eTable 5. Three-year overall survival of patients stratified by histologic pattern group and size of lung adenocarcinoma eTable 6. Association of guideline standard adherence with survival in patients stratifid by histologic pattern group and size eTable 7. Results of inverse probability of treatment weighted multivariable Cox regression eTable 8. Summary of sensitivity analyses eTable 9. Exploratory comparison of two guideline standards in patients with HGNL group adenocarcinoma

Supplement 3. Data Sharing Statement
