# Supplementary material for: Lymph Node Dissection Guideline Adherence and Survival in Patients With T1N0M0 Lung Adenocarcinoma
Source: JAMA Oncol. 2026 Jan 15;12(3):266–74. doi: 10.1001/jamaoncol.2025.5924 (PMC12809417; doi:10.1001/jamaoncol.2025.5924)
Supplement: Supplement 1. — eMethods eResults eFigure 1. Diagram of the study population selection steps eFigure 2. Survival curves by histologic pattern group of lung adenocarcinoma eFigure 3. Survival curves by compliance to lymph node dissection guideline eFigure 4. Directed acyclic graph eFigure 5. Absolute standardized mean difference before and after weighting eFigure 6. Histograms of stabilized weights eReferences [file jamaoncol-e255924-s001.pdf]

Supplemental Online Content

Li R, Wang P, Zhang H, et al. Lymph node dissection guideline adherence and survival in patients with T1N0M0 lung adenocarcinoma. *JAMA Oncol.* Published online January 15, 2026. doi:10.1001/jamaoncol.2025.5924

eMethods

eResults

eFigure 1. Diagram of the study population selection steps

eFigure 2. Survival curves by histologic pattern group of lung adenocarcinoma

eFigure 3. Survival curves by compliance to lymph node dissection guideline

eFigure 4. Directed acyclic graph

eFigure 5. Absolute standardized mean difference before and after weighting

eFigure 6. Histograms of stabilized weights

eReferences

This supplemental material has been provided by the authors to give readers additional information about their work.

© 2026 Li R et al. *JAMA Oncology*

eMethods

Data description

T and N categories, and TNM stages were determined according to the eighth edition of the TNM classification for lung cancer. Pathologic stage IIIB (n = 37) was combined with stage IIIA into stage III due to limited sample size.

Pre-operative positron emission tomography/computed tomography (PET/CT) was defined as the PET-CT for body areas including chest within 90 days before the date of surgery.

The data of pre-operative endobronchial ultrasound (EBUS) or mediastinoscopy, and post-operative adjuvant therapy, were absent in the present database.

Missing values

Missing values in surgery method and lung resection were multiply imputed using chained equations implemented in the "mice" package. The key covariates including survival status, age, sex, smoking, histologic pattern group, year of surgery, lobe-of-origin and center were used for imputation process. Ten imputations were performed to achieve good convergence.

Statistical analysis

Proportional hazard assumptions were examined by Schoenfeld test. Multivariable Cox proportional hazard regression models were performed within each imputed dataset to estimate hazard ratio (HR) and 95% confidence intervals (CI). These models included age (as continuous), sex (female and male), year of surgery (as categorical), smoking history (never and current/ever), surgery method (open surgery, video-assisted thoracoscopy surgery[VATS], and robotic-assisted thoracoscopy surgery[RATS]), lung resection extent (lobectomy, segmentectomy, wedge resection, and extensive resection), lobe-of-origin (right upper lobe, right middle lobe, right lower lobe, left upper lobe and left lower lobe), and a hospital-specific gamma-distributed shared-frailty term to account for hospital-level clustering. Directed cyclic graph was used to help determine the variables included in the Cox regression models (eFigure 4). Efron's method was applied to handle tied events. Results from each multiply imputed dataset were pooled via Rubin's rules.

E-values were estimated according to methods proposed by VandaWeele *et al*[1] to quantify the robustness of results against unmeasured confounders. Concordance index was calculated for models. Good calibration of Cox regression models was examined by visual check of calibration curves with slope close to 1. Absolute risk difference and number needed to treat (NNT) was calculated using method proposed by Altman *et al*.[2]

Events per variable (EPV) was calculated as proposed by Peduzzi *et al*[3]. For analysis with EPV < 10, a bootstrap 95% CI for HR obtained from 1000 bootstrap replicates was also supplemented. For results from analysis with EPV <10, we propose to interpret them as exploratory only and remind the readers of potential inaccuracy of statistic estimation.

Inverse probability of treatment weighting

Inverse probability of treatment weighting (IPTW) was performed. Propensity score (PS) was calculated using logistic regressions including age, sex, smoking history, surgery method, lung resection extent, lobe of origin, year of surgery and center. Stabilized weights were calculated from PS and were examined for extreme outliers by histograms. Satisfying balance of covariates were defined as

© 2026 Li R et al. *JAMA Oncology*

standardized mean difference < 0.1 after weighting. Weighted multivariable Cox regression models with same variables in the formal analysis (except for center which was now directly included) was performed to estimate HR and 95%CI. Propensity score estimation, weighting, HR and 95%CI estimation were first performed within each imputed dataset. Results were then pooled via Rubin's rules.

Sensitivity analysis

Various sensitivity analyses were performed. (1) A competing risk regression was performed to estimate cancer-specific HRs and 95%CIs associated with guideline-adherent LN dissection. (2) A lobectomy-only sensitivity analysis was conducted in patients who underwent lobectomy to ensure that the results were not driven by particular surgery setting. (3) Analyses were conducted stratifying the study population by surgery volume of admission center. As the association between surgery volume and outcome has been extensively evidenced[4-6], we stratified included centers into high-volume (>1000 lung cancer surgeries per year, 3 centers), middle-volume (100-1000 surgeries per year, 6 centers) and low-volume (<100 surgeries per year, 10 centers). (4) Analyses were conducted stratifying the study population by lobe-of-origin to explore potential lobe-specific effect. (5) A truncated-weights sensitivity where stabilized weights calculated from IPTW procedure for each imputed dataset were truncated at 99% quantile. Truncated stabilized weights were used in weighted multivariable Cox regression models to calculate HR and 95% CI on each imputed dataset. Results were pooled via Rubin's rules. (6) A doubly robust augmented inverse-probability-weighted (AIPW) analysis including same variables as described in the IPTW procedure was performed using "coxAIPW" package on each imputed dataset. Results were pooled via Rubin's rules. (7) A tipping-point analysis was performed to determine the effect required for a hypothetical unmeasured confounder associated with poorer prognosis to render results in overall HGNL group nonsignificant. The unmeasured confounder we considered in this study was poor performance status (Eastern Cooperative Oncology Group [ECOG] >= 1). We assumed the proportion of patients with poor performance status according to previous publications[7,8], and according to the fact that all included patients were medically tolerant to a major thoracic surgery. The relationship between the hypothetical unmeasured confounder and overall survival required to render the 95%CI of HR overlapping with null was estimated using "tipr" package.

eResults

Follow-up

All included patients were surveyed with valid follow-up data through harmonization of multiple data sources during the last round of follow-up performed between September 2022 and December 2022. Of the 798 (2.9%) patients who deceased at the end of follow-up, 105 (0.38%) deceased from other causes than lung cancer.

Inverse probability of treatment weighting

Good balancing of baseline variables were achieved with absolute standardized mean difference < 0.1 (eFigure 5). After IPTW, multivariable Cox regression models revealed no association between survival and adherence to the "3+1" standard (HR, 0.95; 95%CI 0.92-1.45), or the 6-station standard (HR, 0.60; 95%CI 0.26-1.40) among patients with LepNH group adenocarcinoma. Associations between survival benefit and adherence to the "3+1" standard (HR, 0.83; 95%CI 0.70-0.98; E-value, 1.71), or the 6-station

© 2026 Li R et al. *JAMA Oncology*

standard (HR, 0.64; 95%CI 0.46-0.89; E-value, 2.49) were observed among patients with HGNL group adenocarcinoma. Detailed HRs, 95% CIs and P values are provided in eTable 7.

Sensitivity analysis

The results of sensitivity analyses are summarized in eTable 8. The competing risk regression revealed that among patients with LepNH group adenocarcinomas, no association between cancer-specific survival and adherence to the “3+1” standard (HR, 0.85; 95% CI, 0.59-1.22), or the 6-station standard (HR, 0.70; 95% CI 0.34-1.45) was observed. Among patients with HGNL group adenocarcinoma, adherence to the “3+1” standard (HR, 0.82; 95% CI, 0.69-0.97; E-value, 1.75), or the 6-station standard (HR, 0.56; 95% CI 0.39-0.81; E-value, 2.96) was associated reduced lung cancer-specific death.

In analysis limiting study population to those who underwent lobectomy, no association was observed between survival benefit and adherence to the “3+1” standard (HR, 0.85; 95% CI, 0.58-1.25), or the 6-station standard (HR, 0.55; 95% CI 0.25-1.20) among patients with LepNH group adenocarcinomas. Association between significant survival benefit and adherence to the “3+1” standard (HR, 0.75; 95% CI, 0.64-0.90; E-value, 1.98), or the 6-station standard (HR, 0.54; 95% CI, 0.38-0.77; E-value, 3.10) was observed among patients with HGNL group adenocarcinoma.

When stratified by surgery volume of admission center, 16497 patients underwent surgery in high-volume centers, 4183 in middle-volume centers and 6511 patients in low-volume center. The significant association between standard adherence and survival benefit in patients with HGNL group adenocarcinoma persisted in most subgroups. In some subgroups, the association became marginally significant, probably due to limited sample size after stratification. No extreme outlier HR, or HR of opposite direction were observed.

When stratified by lobe-of-origin, the significant association between standard adherence and survival benefit in patients with HGNL group adenocarcinoma persisted in most subgroups. The association became only marginally significant in some groups probably due to limited sample size after stratification. No extreme outlier HR, or HR of opposite direction are observed.

Stabilized weights from the IPTW procedure were checked for extreme outliers by weights histograms (eFigure 6). The vast majority of weights lied around one but outliers did exist. Weights for each dataset were truncated at 99% quantile in the truncated-weight sensitivity analysis. Analyses using truncated stabilized weights revealed that among patients with LepNH group adenocarcinoma, no association between survival and adherence to the “3+1” standard (HR, 0.87; 95%CI, 0.60-1.27), or the 6-station standard (HR, 0.62; 95%CI, 0.29-1.33) was observed. Among patients with HGNL group adenocarcinoma, association between survival benefit and adherence to the “3+1” standard (HR, 0.83; 95% CI, 0.70-0.98; E-value, 1.71), or the 6-station standard (HR, 0.63; 95% CI, 0.46-0.87; E-value, 2.54) was observed.

In augmented inverse-probability-weighted analysis, results similar to those from IPTW were observed. Among patients with LepNH group adenocarcinoma, no association between survival and adherence to the “3+1” standard (HR, 0.99; 95%CI, 0.82-1.20), or the 6-station standard (HR, 1.25; 95%CI, 0.98-1.60) was observed. Among patients with HGNL group adenocarcinoma, association between survival benefit and adherence to the “3+1” standard (HR, 0.76; 95% CI, 0.69-0.83; E-value, 1.98), or the 6-station standard (HR, 0.74; 95% CI, 0.62-0.87; E-value, 2.06) was observed.

© 2026 Li R et al. *JAMA Oncology*

In the tipping-point analysis, assuming a prevalence of poor performance status (ECOG >=1) of 5% in the adherence group and 10% in the non-adherence group, the HR for the hypothetical unmeasured confounder would need to be 2.1 or more to render the association between survival benefit and adherence to the "3+1" standard in HGNL group nonsignificant, and 6.2 or more to render the association between survival benefit and adherence to the 6-station standard in HGNL group nonsignificant. These results supported the robustness of our results to unmeasured confounders.

© 2026 Li R et al. *JAMA Oncology*

eFigure 1 Diagram of the study population selection steps

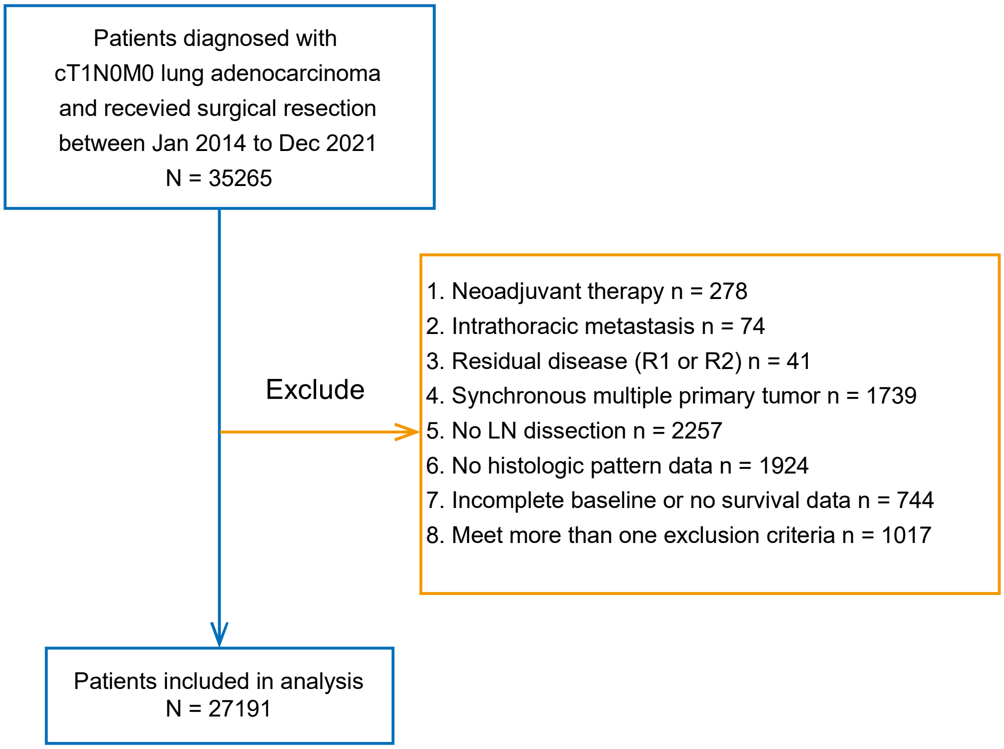

© 2026 Li R et al. *JAMA Oncology*

eFigure 2 Survival curves by histologic pattern group of lung adenocarcinoma

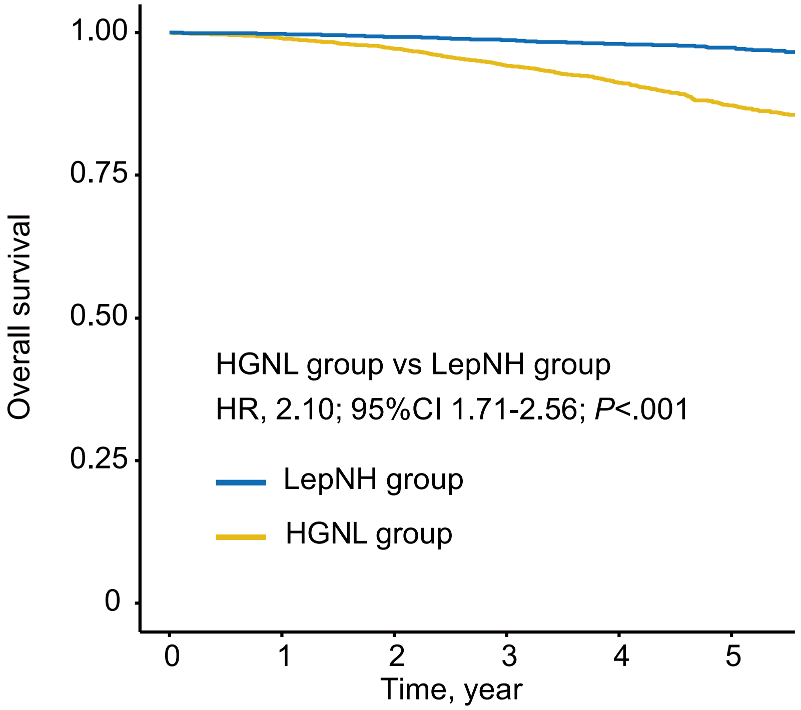

Number at risk

LepNH group	13369	10157	6932	3344	1755	1168
HGNL group	13822	10451	7281	3864	2067	1333

© 2026 Li R et al. *JAMA Oncology*

eFigure 3 Survival curves by adherence to lymph node dissection guideline

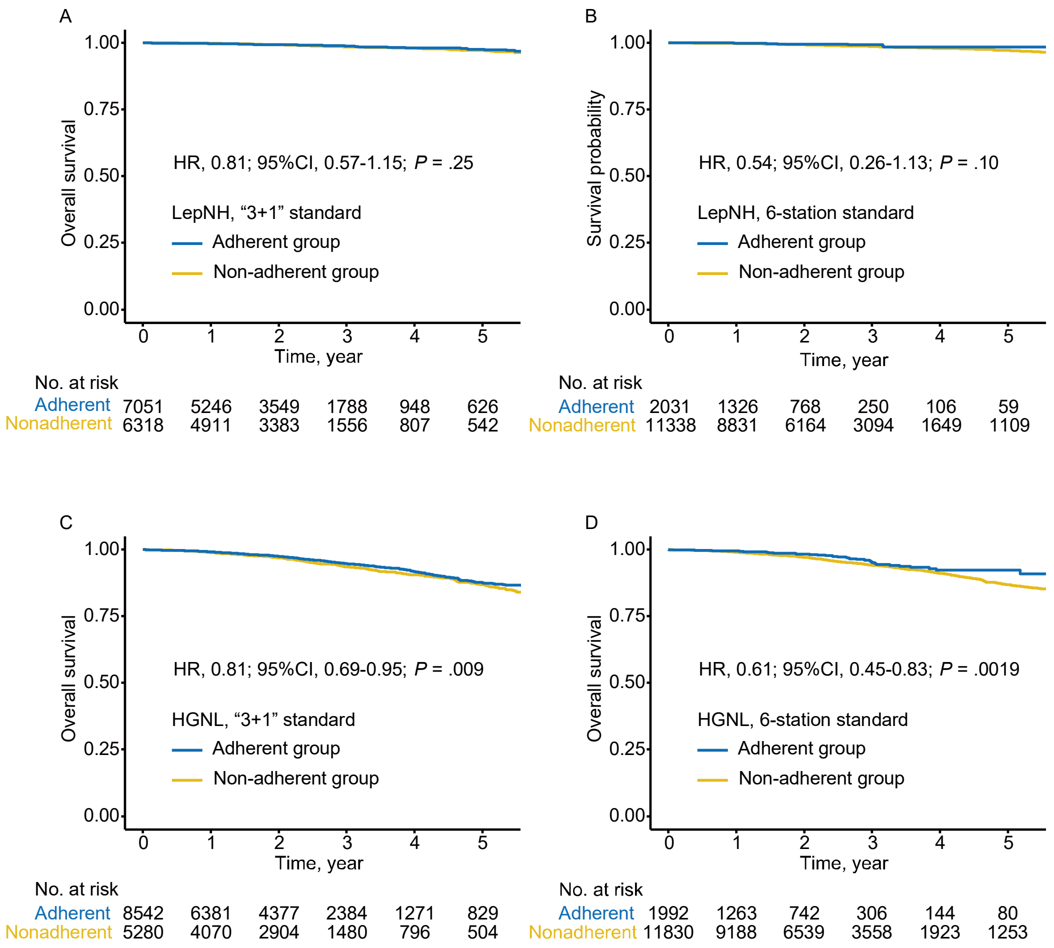

Kaplan-Meier curves for overall survival are plotted for patient with (A) LepNH group adenocarcinomas by adherence to the "3+1" standard; (B) LepNH adenocarcinomas by adherence to the 6-station standard; (C) HGN L group adenocarcinomas by adherence to the "3+1" standard; (D) HGN L group adenocarcinomas by adherence to the 6-station standard.

© 2026 Li R et al. *JAMA Oncology*

eFigure 4 Directed acyclic graph

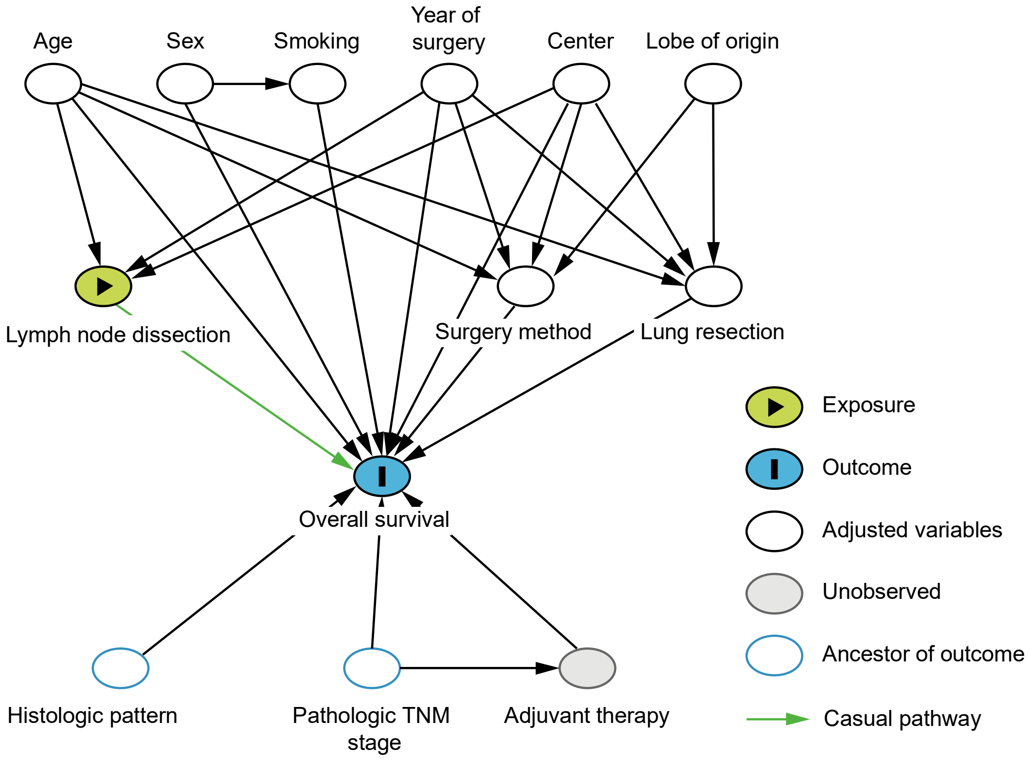

Directed acyclic graph identifying confounders included in the multivariable Cox regression modesl.

© 2026 Li R et al. *JAMA Oncology*

eFigure 5 Absolute standardized mean difference before and after weighting

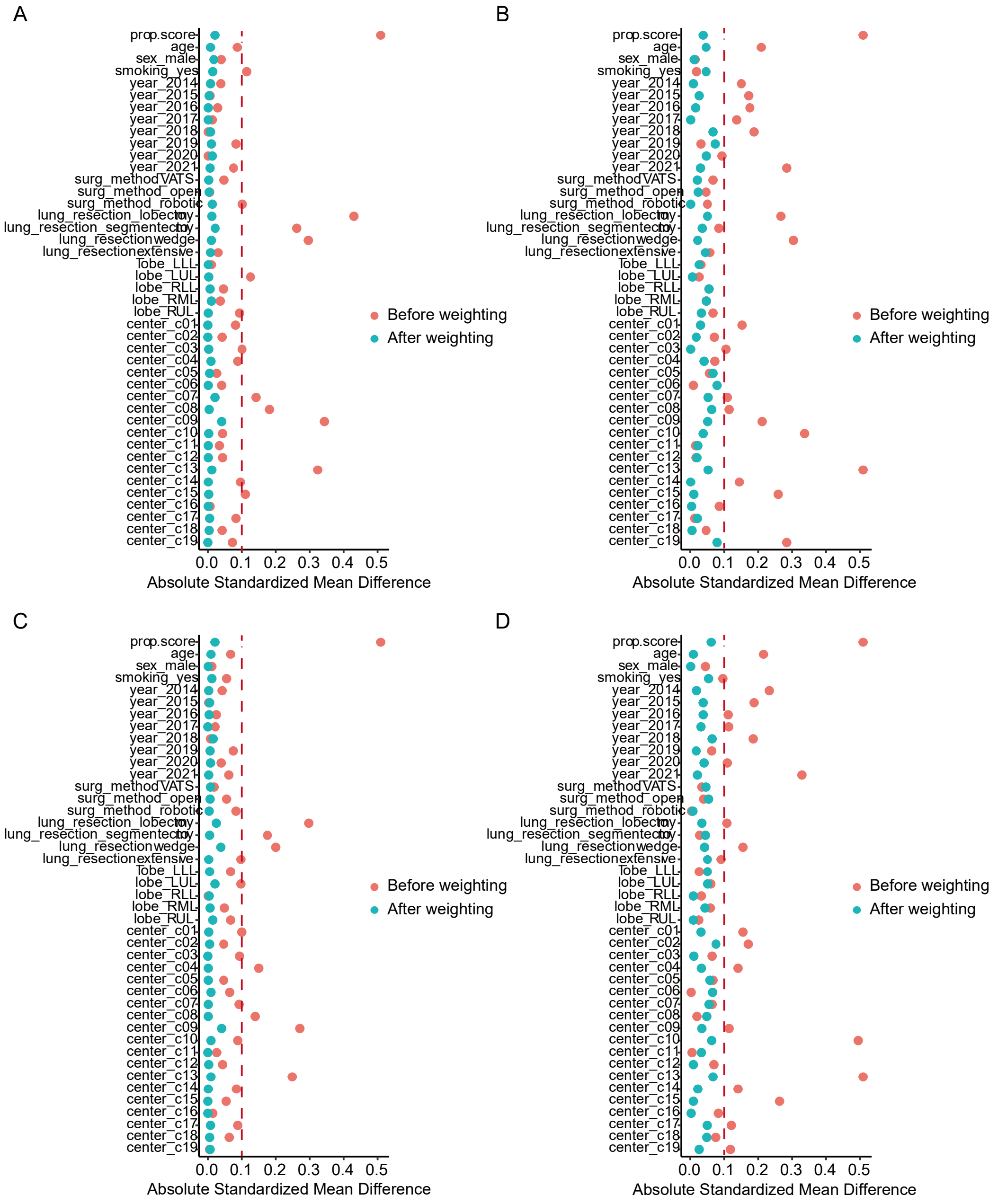

Plot summary for absolute value of standardized mean di fference before and after inverse probability of treatment weighting in (A) patients with LepNH group adenocarcinoma, stratifiey by adherence to the “3+1” standard; (B) patients with LepNH group adenocarcinoma, stratifiey by adherence to the 6-staion standard; (C) patients with HGNL group adenocarcinoma, stratifiey by adherence to the “3+1” standard; (D) patients with HGNL group adenocarcinoma, stratifiey by adherence to the 6-station standard.

© 2026 Li R et al. *JAMA Oncology*

eFigure 6 Histograms of stabilized weights

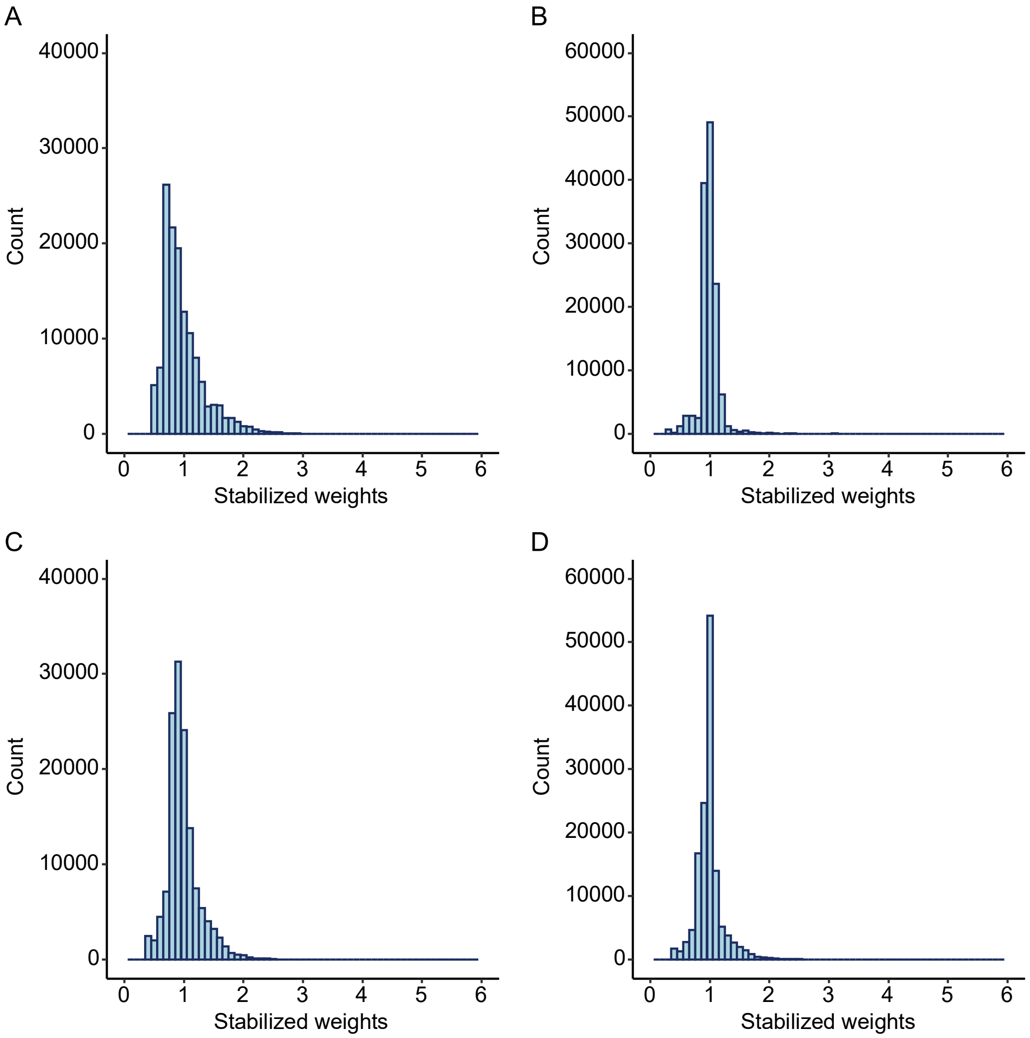

Stabilized weights in patients (A) with LepNH group adenocarcinoma by adherence to the “3+1” standard; (B) with LepNH group adenocarcinoma by adherence to the 6-station standard; (C) with HGN L group adenocarcinoma by adherence to the “3+1” standard; and (D) with HGN L group adenocarcinoma by adherence to the 6-station standard, are plotted as histograms. Weights from ten imputed datasets for each comparison were plotted together in each panel.

© 2026 Li R et al. *JAMA Oncology*

eReferences

1. VanderWeele TJ, Ding P. Sensitivity Analysis in Observational Research: Introducing the E-Value. *Annals of Internal Medicine*. 2017;167(4):268-274. doi:10.7326/m16-2607
2. Altman DG, Andersen PK. Calculating the number needed to treat for trials where the outcome is time to an event. *BMJ*. 1999;319(7223):1492. doi:10.1136/bmj.319.7223.1492
3. Peduzzi P, Concato J, Kemper E, Holford TR, Feinstein AR. A simulation study of the number of events per variable in logistic regression analysis. *Journal of Clinical Epidemiology*. 1996;49(12):1373-1379. doi:10.1016/S0895-4356(96)00236-3
4. Panageas KS, Schrag D, Riedel E, Bach PB, Begg CB. The Effect of Clustering of Outcomes on the Association of Procedure Volume and Surgical Outcomes. *Annals of Internal Medicine*. 2003/10/21 2003;139(8):658-665. doi:10.7326/0003-4819-139-8-200310210-00009
5. Levaillant M, Marcilly R, Levaillant L, et al. Assessing the hospital volume-outcome relationship in surgery: a scoping review. *BMC Medical Research Methodology*. 2021;21(1)doi:10.1186/s12874-021-01396-6
6. Kim BR, Sohn JY, Jang EJ, Jo J, Lee H, Ryu HG. Hospital case-volume and mortality after lung cancer surgery: A population-based retrospective cohort study. *Lung Cancer*. 2022;169:61-66. doi:10.1016/j.lungcan.2022.05.016
7. Saji H, Okada M, Tsuboi M, et al. Segmentectomy versus lobectomy in small-sized peripheral non-small-cell lung cancer (JCOG0802/WJOG4607L): a multicentre, open-label, phase 3, randomised, controlled, non-inferiority trial. *Lancet*. Apr 23 2022;399(10335):1607-1617. doi:10.1016/S0140-6736(21)02333-3
8. Suzuki K, Watanabe S-i, Wakabayashi M, et al. A single-arm study of sublobar resection for ground-glass opacity dominant peripheral lung cancer. *The Journal of thoracic and cardiovascular surgery*. 2022;163(1):289-301.e2. doi:10.1016/j.jtcvs.2020.09.146

© 2026 Li R et al. *JAMA Oncology*
