# Supplementary material for: Lymph Node Dissection Guideline Adherence and Survival in Patients With T1N0M0 Lung Adenocarcinoma
Source: JAMA Oncol. 2026 Jan 15;12(3):266–74. doi: 10.1001/jamaoncol.2025.5924 (PMC12809417; doi:10.1001/jamaoncol.2025.5924)
Supplement: Supplement 3. — Data Sharing Statement [file jamaoncol-e255924-s003.pdf]

Data Sharing Statement

Li. Lymph Node Dissection Guideline Adherence and Survival in Patients With T1N0M0 Lung Adenocarcinoma. *JAMA Oncol*. Published January 15, 2026.
doi:10.1001/jamaoncol.2025.5924

Data
Data available: No
